# Fertility Intentions for a Second Child and Their Influencing Factors in Contemporary China

**DOI:** 10.3389/fpsyg.2022.883317

**Published:** 2022-05-19

**Authors:** Mingming Li, Xinxin Xu

**Affiliations:** ^1^Department of Economics and Business, Central European University, Vienna, Austria; ^2^Doctoral School of International Relations and Political Science, Corvinus University of Budapest, Budapest, Hungary

**Keywords:** fertility intentions, two-child policy, machine learning, artificial neural network, random forest, XG-boost, logistic regression

## Abstract

Although the Chinese government has shifted from a one-child policy to a two-child policy (allowing a couple to have up to two children) since 2016 in response to the aging population, the policy results have been unsatisfactory. This is the first paper to systematically investigate the factors influencing residents’ intentions to have a second child. The research focuses on the perspective of individual, family, and social characteristics based on the Chinese General Social Survey (CGSS) from 2017 to 2018. Three machine learning methods are used in conjunction with logistic regression to reveal that the intention of having a second child increases heavily with age, more siblings in the family of origin, and better health. The family income, which is currently the focus of the literature and is statistically significant, is only sixth most important. This study further reveals differences between genders: Women with a lower level of education and religious beliefs prefer to have a second child, whereas for men, non-agricultural hukou and marriage are the position factors. The results of this study also illustrate the importance of future research focusing on the relationship of individuals to their family of origin and districts.

## Introduction

The family planning policy is the basic state policy of China, and its strict implementation has driven a dramatic shift in China’s population growth pattern. Between the 1970s and the mid- to late-1990s, China’s total fertility rate (TFR) dropped sharply, from 5.81 children per family to approximately 1.80. By 2015, the National Bureau of Statistics released data on the 1% population sample survey, which indicated that China had entered a stage of severe under-population. Once the TFR falls below 1.5, the society falls into a “low-fertility trap,” and it is difficult to return to normal levels of generational replacement over a longer period of time (Lutz and Skirbekk, 2005). When China entered an aging society in 2000, there were about 130 million people over the age of 60, accounting for 10.2% of the total population; by the end of 2019, the number of people over the age of 60 had risen to nearly 254 million, representing 18.1% of the total population.

The alarming trend of couples having fewer children, combined with an aging population, has led to the disappearance of the demographic dividend, posing a significant challenge to China’s economic and social development. To address the challenges of continued low fertility and an aging population, in January 2016 the Chinese government endorsed the birth of two children per couple. There have been mixed views among scholars on this policy. Proponents argue that the policy will lead to a great increase in TFR and slow down the process of population aging (Zhai et al., 2014). However, other researchers take the opposite view, citing the high cost of raising children and sense of gender equality (Wang and Zhang, 2012; Jun, 2015). Today it has become a social consensus that the two-child policy does not work well in China because the TFR did not increase as expected. With the change in fertility policy, the fertility incentives faced by families are bound to change. Therefore, it is important to clarify the factors influencing couples’ willingness to have a second child so that we can promote an effective intervention policy that will optimize the demographic structure. At the same time, the study on the fertility intentions of second child can help to design the future progressive liberalization of population policy (three-child policy or even full liberalization).

Considering the above realities of China’s socioeconomic situation, this paper seeks to examine the following core questions: (1) What factors have important impacts on couples’ willingness to have a second child in China? (2) What are the differences between men and women in terms of their willingness to have a second child? Specifically, this paper uses data from the Chinese General Social Survey (CGSS) to analyze the relationships between the fertility intentions of different cohorts and the variables impacting those intentions. The paper uses three machine learning methods—artificial neural network (ANN), random forest (RF), and XG-boost—to systematically identify the important factors; next, a logistic regression model is used to investigate the mechanisms of the factors that influence the willingness to have a second child.

## Literature Review

Scholars have conducted many studies investigating the factors that influence fertility outcomes, and that body of research can generally be categorized into micro, meso, and macro perspectives. At the micro level, these can be further divided into psychological decision-making dimensions, such as the desire to have children (Ajzen, 1991; Spéder and Kapitány, 2009; Morgan and Bachrach, 2011); partnership dimensions, such as marital relationships and the division of household labor (Corijn and Klijzing, 2013); individual socioeconomic dimensions, such as income and human capital, including education and employment (Kravdal, 1992; Becker et al., 1999); and the influence of underlying biological and genetic traits (Kohler et al., 1999; Kohler and Rodgers, 2003). The meso level can be divided into the social-interaction dimension, including personal networks and social learning (Montgomery and Casterline, 1996; Bernardi, 2003); the place-of-residence dimension, which focuses on the heterogeneity of regions (Hank, 2001, 2002; Caltabiano et al., 2009); and the social capital dimension, which includes goods as well as information, money, work capacity, influence, power, and positive help (Granovetter, 1973; Bourdieu and Richardson, 1986; Coleman, 1988). At the macro level, the dichotomy between “economy and culture” (Baizán et al., 2004) dominates and considers economic and employment trends (Butz and Ward, 1979; Macunovich, 1996) along with values and culture (Lesthaeghe and Van de Kaa, 1986; Pfau-Effinger, 1999).

Family policies and welfare systems can strongly influence fertility outcomes. As the birthplace of the modern welfare system, European countries have implemented complementary family policies such as paid leave entitlement, childcare services, and financial transfer to avoid a continuous decline in population by extending state involvement to the family-reproduction sphere (Gauthier, 2002). This state involvement in the family domain has prevented a steady decline in the birth rate in recent years. Although many studies have attempted to capture the causality between patterns of demographic transitions and types of family policies and welfare regimes (Gauthier, 2007; Kalwij, 2010), encouraging fertility is a very complex, systemic ambition, and there are significant differences in the values, programs, and support of fertility policies depending on the welfare regimes, resulting in very different fertility outcomes across countries.

In terms of the statistical methods used to study fertility, there are currently two focuses of research. The first is the analysis of causality and endogeneity, such as the link between the field of education and age at first birth at the micro level (Lappegård, 2002), the problem of correctly identifying social-interaction effects at the meso level (Manski, 1993, 1995), and the difficulty of distinguishing the effects of policies from other factors—observable or unobservable—at the macro level. The second focus is on the prediction of fertility outcomes by machine learning, which has been very poorly covered in the literature. Studies from the United States, India, Indonesia, and other regions (Bandyopadhyay and Chattopadhyay, 2006; Otoom et al., 2019; Riiman et al., 2019; Nyoni et al., 2021) have used different machine learning algorithms like regression, decision trees, k-nearest neighbors, and ANN, as well as other ensemble methods like bagging and boosting, to determine the population. This relied on the availability of historical demographic data like population, fertility, mortality, and life expectancy. However, the current studies have not focused on estimating population and TFR in a data-constrained environment and with a lack of international comparison.

Since the population policy is unique to China and has been implemented since 2016, scholars have conducted extensive research on second-child fertility. Chen et al. (2019) conduct a comprehensive analysis using stepwise regression and found that household economic and health risks significantly influence the intention to have a second child. Zhou and Guo (2020) use multilevel regression to find that men, the younger and wealthier, ethnic minority, and rural populations are more likely to have a second child.

Scholars have also attempted to focus their research on one perspective. Economic factors are the focus of current research, with Lan (2021) demonstrating that women with better socio-economic status and those who were born into better-off families show a relatively strong desire to have children, and Shen and Jiang (2020) investigating highly educated women and finding that their fertility choices are the result of the intersection of state policy interventions and career choices. Urban-rural differences have captured the attention of many scholars as well. Using the Blinder-Oaxaca decomposition technique, Zhou and Guo (2021) find that both education and son preference play a prominent role in explaining the willingness to have a second child in rural areas, and Li et al. (2022) note that women face the dilemma of having children or seeking employment stability, and that this effect is stronger for urban than rural women. In China today, small families are preferred, and women also have more of a voice in family issues and decision-making (Ding and Hesketh, 2006), so scholars have also studied gender equality and fertility intentions. Bao et al. (2017) and Li and Jiang (2019) find that the more equal gender role attitudes are, the more women with more economic and family/social resources are more likely to have a second child, while women who work in the non-agricultural sector and have higher decision-making power in the household are less likely to have a second child. The influence of siblings has also intrigued scholars, and the number of siblings has been shown a significant predictor of women’s fertility intentions (Zhang C. et al., 2021). The loneliness experienced by only children during childhood and adulthood leads most of them to believe that having siblings is better than being single (Lan, 2021). However, Zhang L. et al. (2021) argue that sibship size may also have negative direct effect presumably due to sibling competition for intergenerational support.

Scholars have sought to further analyze it also through qualitative analysis methods such as interviews. Using qualitative data from 53 urban parents in China, Peng (2020) suggests that the fertility decision to have a second child is an ongoing bargaining process rooted in the life course rather than an isolated family event. In addition, attitudes and behaviors toward fertility in China are rooted in Confucian philosophies and traditions of ancestor worship and may also be influenced by religious notions of fertility (Logan et al., 2019).

Therefore, the status of fertility studies in China needs to be improved in three aspects. First, the data need to be updated. Due to the delay in data release, the existing research mainly comprised survey data through 2015. However, the data from 2017 to 2018 represent important improvements in the content of the questionnaire, with more informative content, a more reasonable design, and higher data quality. Second, the methodology needs to be improved. Most of the existing studies used a single model for empirical evidence, such as probit or logistic regression. If the relationship between the independent and dependent variables does not conform to the regression form, it will not be extracted by the model, and the important variables can be easily missed. This results in one-sided empirical results, which cannot achieve the desired comprehensive, systematic, and integrated test. Third, the generalizability of research findings needs to be improved. The existing studies focused on the local population and cannot grasp the relationship between the influencing factors and fertility from the overall population.

In summary, inspired by the micro-meso-macro classification and applying it to the specific national situation of China and the characteristics of CGSS data (mainly on micro and meso levels), this study focuses on three characteristics and proposes four hypotheses.

*Hypothesis 1: Individual characteristics (e.g., gender, age, ethnicity, health, and education level) will affect fertility intentions.*

*Hypothesis 2: Family characteristics (e.g., family income and number of siblings) will affect fertility intentions.*

*Hypothesis 3: Social Characteristics (e.g., location, religion, health insurance, and hukou) will affect fertility intentions.*

*Hypothesis 4: The above three groups of characteristics will have different effects on the fertility intentions of males and females.*

The subsequent portion of this paper is structured as follows: The third section introduces the machine learning models that will be used in this paper and briefly describes the data sources and classifications; the fourth section introduces the data and methodology; the fifth section describes an empirical analysis in which the importance of different factors is analyzed by machine learning and quantified and explained using logistic regression. Finally, a discussion and summary are presented for the benefit of future research.

## Materials and Methods

### Machine Learning Methods

The connection of the variable “willingness toward fertility” to different independent variables is always complex and shifting, and the relationship between variables and willingness toward fertility is non-linear. Because machine learning methods can fit non-linear information as well as linear data, this paper adopts three common machine learning methods to identify the influencing factors: ANN, RF, and XG-boost, and it adopts a logistic regression model to study the mechanism of those influencing factors of willingness toward fertility. Because this paper adopts machine learning, data mining, and data-driven research methods and ideas, and starts from data and objective reality rather than *a priori* assumptions, the relevant variables are selected to reflect the respondent’s basic personal information, work information, and health information. This method makes the profile more detailed, the behavior measurement more accurate, and the empirical conclusion more comprehensive, systematic, and effective.

At the same time, these three machine learning methods can support each other to obtain more scientific conclusion. First, from the perspective of optimization methods, the ANN is a local search-optimization method, which may fall into local extremes instead of global optima, thus affecting the overall training effect. The RF technique is a random method to build a forest, which adopts the principle of “minority follows majority” to perform integrated discrimination and ensure the overall optimum. The XG-boost is a method to grow a forest by continuously splitting feature variables to ensure the overall optimum. XG-boost grows the tree by splitting the feature variables continuously and relearns every tree generated to continuously improve the learning quality and dynamically approach the overall optimum. Second, from the viewpoint of applicability, RF has the tendency of overfitting when the data is noisy; XG-boost can effectively prevent the overfitting problem by introducing penalty terms. The ANN has strong fault tolerance and can work normally even after local damage. Finally, in terms of computational efficiency, XG-boost pre-sorts the nodes features before iteration and iterates through them to select the optimal partition point, which is a greedy algorithm that takes a longer amount of time when the data volume is large.

#### Artificial Neural Network Model

**FIGURE 1:**
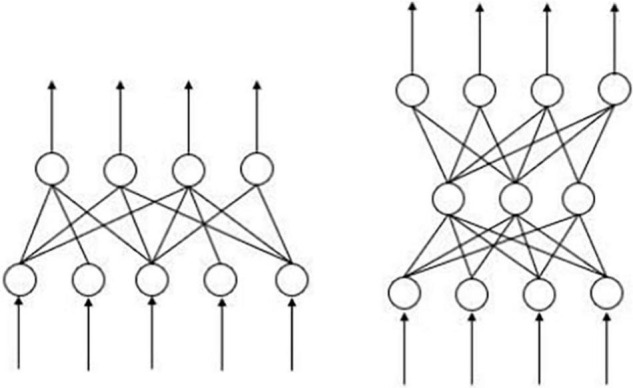
Common two-layer and three-layer ANN models.

The ANN is an abstract computational paradigm modeled after the human brain that consists of interconnected neurons, i.e., processing units, which simulate the human brain’s thinking for computational modeling. Figure 1 shows the common two-layer and three-layer ANN. The B-P ANN model used in this paper is a kind of feed-forward ANN, which has many advantages. First, it has a strong non-linear mapping capability. As many of the independent and dependent variables in the underlying data are non-linear, and the strength and form of the relationships are unknown, the ANN can solve this problem well. Second, the ANN has outstanding self-learning and self-adaptive capabilities. During the training process, the model can automatically extract the data rules between the input data and the output data and write down the learned “rules” through the weights. This allows the ANN to spontaneously learn the connections between the data and objectively reflect these connections. Third, it is highly fault-tolerant, ensuring that the global training results do not deviate significantly when local or partial neurons are damaged and that the system can still function properly even after local damage.

#### Random Forest Model

The RF model is an RF technique that contains several decision trees with high prediction accuracy that are weakly correlated or even uncorrelated. Based on the classification results of these decision trees, the principle of “majority rule” is applied to make a comprehensive, integrated discrimination. Random forest is a combined prediction model that contains multiple decision trees. As a common method of machine learning, RF has wide applicability due to its simple and easy-to-understand modeling principle, and it offers several advantages. First, RF can handle many input variables, i.e., it can handle high-dimensional data. Second, the RF uses error estimation for errors, which makes the model generalize well. For unbalanced data, the RF can balance the errors and maintain the accuracy of the model even when the features are missing. Finally, since the RF is composed of decision trees, and these decision trees are independent of each other, each tree can be processed in parallel, which makes the model training fast. Through the idea of integration, the integrated discriminative classification is performed based on the classification results of all trees.

#### XG-Boost Classification

The basic purpose of the XG-boost algorithm is to keep generating the tree and to grow the tree by continuously splitting the feature variables. Each time a tree is generated, a new function is relearned to fit the residuals of the last prediction, which improves the learning quality and approximates the actual value. Compared with the traditional gradient boosted decision tree (GBDT) algorithm, XG-boost has significant advantages. First, the traditional GBDT algorithm model only uses the information of first-order derivatives in the optimization process, while XG-boost performs the second-order Taylor expansion of the penalty function and uses the information of first-order derivatives and second-order derivatives for optimization, which makes XG-boost’s optimization faster. Second, XG-boost can automatically learn the splitting direction when there are missing values in the sample, and after each iteration, the model assigns the learning rate to the leaf nodes, reduces the weight of each tree, reduces the impact of each tree, and provides better learning space for subsequent training. Finally, XG-boost also supports parallelism but unlike RF, this parallelism is at feature granularity rather than tree granularity. In sum, the XG-boost model can quickly and accurately complete the classification and prediction of data, and the addition of penalty terms can effectively prevent overfitting based on high accuracy.

#### Evaluation Indicators of Results

All the machine learning model results are analyzed based on the confusion matrix, as shown in Table 1. True positives (TP) indicate the number of samples with positive actual results and positive predicted results; false positives (FP) indicate the number of samples with negative actual results but positive predicted results; false negatives (FN) indicate the number of samples with positive actual results but negative predicted results; true negatives (TN) indicate the number of samples with negative actual results and negative predicted results. There are three main associated indexes. (1) Precision rate: Precision = TP/(TP + FP), which indicates the proportion of the actual positive samples among the predicted positive samples. (2) Recall rate: Recall = TP/(TP + FN), which indicates the percentage of predicted positive cases in the sample of actual positive cases. (3) Area under curve (AUC), which indicates the size of the area under the ROC curve. This study also uses machine learning to rank the variables according to their importance from largest to smallest according to the *F*-score.

**TABLE 1:** Confusion matrix.

		Predicted value		
		0	1	Sum
Actual value	0	TN	FP	FP + TN
	1	FN	TP	TP + FN
	Sum	FN + TN	TP + FP	TP + FN + FP + TN

### Logistic Regression

Logistic regression modeling is a multiple regression analysis method used to study the relationship between dichotomous dependent variables and their influencing variables, i.e., it assesses whether an event occurs and what the probability of occurrence is when the influencing variables take on different values.

Assume a vector X = (x1, x2, x3,…, xn) with n independent variables, and assume that the conditional probability *P* (y = 1| x) = *p* is the probability of occurrence of the event in the dependent variable when the independent variables take values. The logistic model can be expressed as:


(1) $$P\left(y=1|x\right)=\pi \left(X\right)=\frac{1}{1+e^{-g\,\left(x\right)}}$$

$f\,\left(x\right)=\frac{1}{1+e^{-g\,\left(x\right)}}$ is called logistic function, *g*(*x*) = ω_1_ + ω_1_*x*_1_ + … + ω_*n*_*x*_*n*_, then the probability of not occurring under condition x is:


(2) $$P\left(y=0|x\right)=1-P\left(y=1|x\right)=1-\frac{1}{1+e^{-g\,\left(x\right)}}=\frac{1}{1+e^{g\,\left(x\right)}}$$

Therefore, the ratio of the probability of an event occurring to the probability of it not occurring is:


(3) $$\frac{P\left(y=1|x\right)}{P\left(y=0|x\right)}=\frac{P}{1-P}\,e^{g\,\left(x\right)}$$

Logistic regression models can be solved iteratively by using the gradient ascent algorithm or by using the Newton-Raphson iteration, and the values of the dependent variable are (0, 1), and after modeling, the probability values of the dependent variable represent the probability of the event. The logistic regression model can predict not only in-sample but also out-of-sample data, and it can compare and test the prediction results.

## Data

### Data Source

The data came from the CGSS questionnaire (resident questionnaire) for two consecutive years—2017 and 2018. The survey is the first nationwide, comprehensive, and continuous large-scale social survey project in China, and it includes 125 counties (districts), 500 streets (townships), 1,000 neighborhood (village) committees, and 10,000 individuals in households. A total of 25,369 samples were obtained from the survey data in 2017 and 2018, and according to the characteristics of the research subjects of this paper, the sample of people of childbearing age between 20 and 50 was selected. The sample was selected according to the characteristics of the research population, and after determining the relevant variables that conform to the research content of this paper, some “missing,” “don’t know,” “indifferent,” “unable to answer,” and “not applicable” were excluded. Ultimately, 15,909 valid samples were obtained.

### Variable Description

In keeping with the purpose of this study, the dependent variable is whether or not respondents are willing to have a second child. Specifically, the questionnaire asks, “How many children would you like to have if there are no policy restrictions?” If the answer is fewer than two (in other words, the respondent does not want to give birth to more than one child), we assign the value “0”; for those who are willing to have two or more children, we assign the value “1.” The independent variables include basic individual characteristics, family characteristics, and social characteristics. Individual characteristics include age, ethnicity, health, and education level. Family characteristics include the respondent’s marital status, family economic level, and the number of people living together in the family. Social characteristics include the respondent’s geographic location, hukou, religious beliefs, and decision about participating in medical treatment insurance. The specific variable descriptions are shown in Table 2.

**TABLE 2:** Independent variable statement.

	Variable	Question	Value assignment
Individual characteristics	Gender	What is your gender?	Male: 1, Female: 0
	Age	What is your date of birth?	For CGSS (2017): 2017–year of birth +1; for CGSS (2018): 2018–year of birth +1
	Ethnicity	What is your ethnicity?	Han: 1, Other ethnicities: 0
	Health status	What do you feel is your current physical condition?	Very unhealthy: 1, relatively unhealthy: 2, average: 3, relatively healthy: 4, very healthy: 5
	Education level	What is your current level of education?	Illiterate: 0, Elementary: 1, Middle: 2, High: 3, Bachelor: 4, Graduate and above: 5
Family characteristics	Marital status	What is your current marital status?	No spouse: 0; first marriage with spouse: 1; remarriage with spouse: 2
	Family income level	Where does your family’s economic status fall in terms of location?	Far below average: 1, below average: 2, average taken: 3, above average taken 4, far above average taken: 5
	Sibling	How many siblings you have?	Take values by the answered number
Social characteristics	District	Which area do you live in?	West: 0, Central: 1, East: 2, Northeast: 3
	Religion	Do you have religious beliefs?	No: 0, Yes: 1
	Health insurance	Do you have health insurance?	No: 0, Yes: 1
	Hukou	What is your hukou status?	Agricultural hukou: 0, Non-agricultural hukou: 1

## Empirical Analysis

### Identification of Factors Influencing Fertility Intention

From the descriptive statistical analysis, the influence of willingness to have two children on different independent variables is complex and variable. That relationship is non-linear, and machine learning methods can fit the non-linear information well. Therefore, this paper adopts the three commonly used machine learning methods previously mentioned in attempting to identify the influencing factors.

#### Artificial Neural Network Modeling

A total of 15,916 samples are included in the base data of this study. The categorical independent variables (such as gender, region, and hukou) are transformed into dummy variables with horizontal signs, and the ordinal independent variables (such as education, health status, and income level) are transformed into factors with numerical signs. This may cause the model to learn the characteristics of those who are willing to have a second child, but not those who are not willing to have a second child, resulting in poor classification results. Therefore, in this paper, we use a down-sampling method to randomly select 3,366 samples of people who are willing to have a second child and obtain a total of 6,732 samples for modeling.

The main purpose of this paper is to separate the data into two categories: those who are willing to have a second child and those who are unwilling to have a second child. Before training, the data are randomly split into a test set and a training set in the ratio of 3:7. In determining the topology of the ANN, a trial-and-error method is used to build a four-layer ANN after trying different ANN structures. The prediction results on the test set are obtained based on the training set model, and the confusion matrix with the actual categories in the test set is shown in Table 3.

**TABLE 3:** Confusion matrix of artificial neural network.

		The actual value in the test set	
		1212	945
Prediction	0	731	348
	1	481	597

It is apparent that the category “willing to have a second child” is applicable to 1,078 people, of whom 597 are judged to be “willing to have a second child,” and 481 are judged to be “unwilling to have a second child.” For the category “unwilling to have a second child,” 731 people are judged to be “unwilling to have a second child,” and 348 are judged to be “unwilling to fertility.” For the “willing to have a second child” category, the precision rate of the model is 597/(597 + 481) = 55.4%, and the recall rate is 597/(597 + 348) = 63.2%. This means that about two-thirds of those who are willing to fertility are judged to be correct, while the model misclassified two-fifths of the sample as “willing to have a second child.” The result of classification of the ANN is acceptable. The independent variables are more effective in distinguishing those who are willing to have a second child, but the model cannot give a numerical measure of which variables are more important for classification. In practice, there are significant differences in the values of “willing to have a second child” and “unwilling to have a second child” in terms of gender, age, health status, education, number of siblings, and region. In conclusion, although the ANN can fit the non-linear relationship between variables well, it cannot measure the importance of each variable, and to remedy this deficiency, this paper uses RF to investigate further.

#### Random Forest Modeling

**FIGURE 2:**
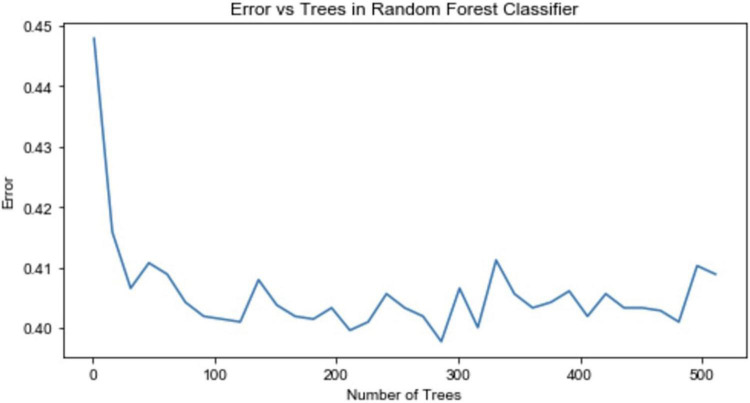
Relationship between the out-of-bag error rate and the number of decision trees.

The same extended 6,732 samples are used to build the RF model. To determine the number of decision trees in the RF, the relationship between the out-of-bag error rate and the number of decision trees is drawn, as shown in Figure 2. When the number of decision trees reaches 286, the error rate reaches its lowest point and stabilizes; therefore, an RF model with 286 decision trees is established.

The model obtained in the training set is applied to the test set, and the prediction results are compared with the actual values of the sample. The classification confusion matrix is shown in Table 4; of the 1,068 people who are willing to have a second child in the test set, 647 are judged as “willing to have a second child” and 421 are judged as “unwilling to have a second child.” The actual number of those “unwilling to have a second child” in the test set is 1,089, among whom 659 are judged as “unwilling to have a second child” and 430 are wrongly judged as “willing to have a second child.” For the “willingness to have a second child” category, the precision rate of the model is 647/(647 + 430) = 60.1%, and the recall rate is 647/(647 + 421) = 60.6%. The RF has lower precision than the ANN, but a higher recall rate.

**TABLE 4:** Confusion matrix of random forest (RF).

		The actual value in the test set	
		1080	1077
Prediction	0	659	430
	1	421	647

The RF classification model can play a vital role in the classification and can prevent the omission of important variables. The results of the importance assessment of the variables are shown in Table 5. Here, the top five most important variables are age, number of siblings, health, education, and district. Age is the most important factor—neither income nor health. The second-most important variable is the number of siblings, which reveals the importance of the family of origin.

**TABLE 5:** Importance of feature variables.

Variable	Importance
Age	1
Sibling	2
Health	3
Education	4
District	5
Income	6
Marriage	7
Hukou	8
Gender	9
Health_insurance	10

#### XG-Boost Modeling

**FIGURE 3:**
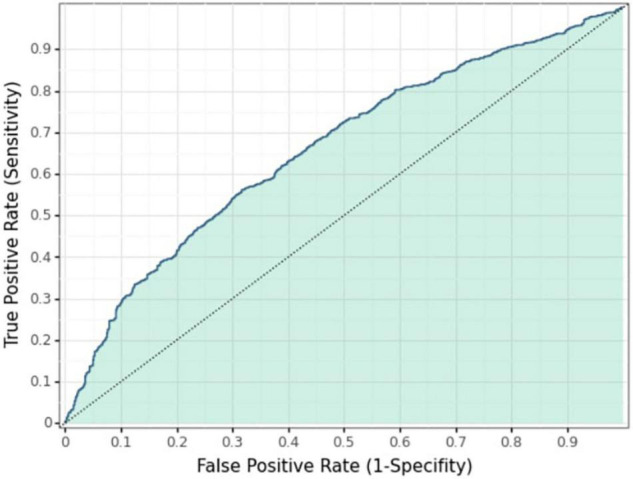
ROC curve.

The values of the XG-boost model at each sample point are obtained with the AUC indicator due to the optimization target and the maximum depth of the number of five. To classify the samples into two categories based on the values of the sample points, an optimal threshold is determined based on the ROC curve (subject operating characteristic curve), which is used as a threshold to segment the samples to achieve the highest accuracy of the classification results. The ROC curves in Figure 3 reflect the classification accuracy obtained with different values of the classification threshold. As demonstrated, the classification results are better when the threshold value is around 0.40.

The best classification result is obtained when the threshold value is 0.40, and the AUC is the largest. The samples less than 0.40 are classified as “unwilling to have a second child,” and those greater than or equal to 0.40 are classified as “willing to have a second child.” The confusion matrix of classification results and actual categories is shown in Table 6. Among the 1,068 people who are actually “willing to have a second child” in the test set, 620 people are judged by the model as “willing to have a second child,” and 448 people are judged by the model as “unwilling to have a second child.” Among the 1,089 people in the test set who are actually “unwilling to have a second child,” 697 are judged by the model as “unwilling to have a second child,” and the remaining 392 are wrongly judged as “willing to have a second child.” The precision rate of the model is 620/(620 + 392) = 61.3%, and the recall rate is 620/(620 + 448) = 58.1% for the category of “willing to have a second child.” The precision rate of the XG-boost model is in between those of the ANN and RF, but the recall rate is the lowest.

**TABLE 6:** Confusion matrix of XG-boost.

		The actual value in the test set	
		1145	1058
Prediction	0	697	392
	1	448	620

**FIGURE 4:**
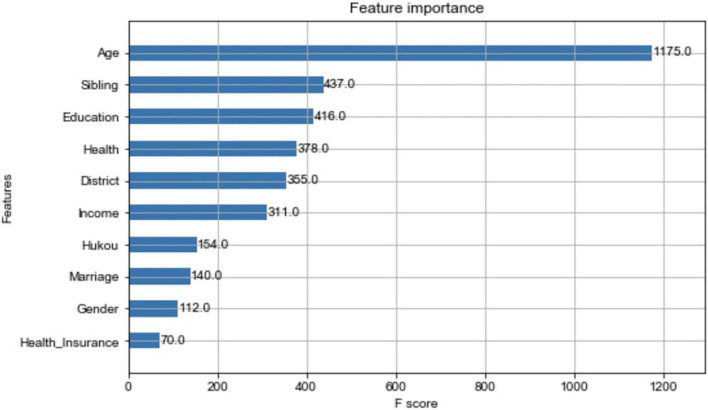
Importance of variables in XG-boost model.

The XG-boost model also examines the importance of variables, and the importance histograms are shown in Figure 4. The XG-boost modeling process plays an important role in age, number of siblings, education, health, and district. Although some of the other variables changed (e.g., gender and marriage), they are consistent with the variables identified in the RF model, and the results of the two models can be corroborated by each other.

#### Comparative Analysis of Empirical Machine Learning Results

To more objectively compare the results of each model, the modeling method of fivefold cross-validation is used, and the data on the average precision rate and average recall rate obtained are shown in Table 7. All of the average precision and average recall rates of the three models are above 60% under the fivefold cross-validation. The ANN has the highest mean precision rate (64.3%), and the RF has the highest recall rate (60.6%) for the category “willing to have two children.” The important variables obtained from the RF and XG-boost models—age, number of siblings, health status, income, and beliefs—are basically the same, although there are very small differences in ranks. Notably, economic level, although important but ranked sixth, is not as important as the previous five items. Therefore, we can validate hypotheses 1, 2, and 3: Individual characteristics, family characteristics, and social characteristics all have an important impact on the intention to have a second child in China.

**TABLE 7:** Comparison of machine learning model results.

Model	B-P ANN	RF	XG-boost
Precision rate	64.10%	61.00%	63.12%
Recall rate	63.64%	59.88%	61.56%

### Logistic Regression

If we use the machine learning classification model, it can only be determined that these variables are significantly related to “willingness to have two children.” The exact quantitative relationship and significance would need to be determined by regression models. Since the dependent variable of the sample is a dichotomous variable, a logistic regression model is used for further empirical study.

#### Logistic Regression of Full Sample

The correspondence parameters and variables after using the logistic regression model are shown in Table 8. Overall, there are more significant coefficients in family and social characteristics than in individual characteristics. For family characteristics, age has a strong effect on the willingness to have a second child, with each additional year of age increasing the willingness to have a child by 0.5%. High school and bachelor’s education, on the other hand, decrease the willingness to have a second child. Gender, health, ethnicity, and other education levels do not have a significant effect on second-child fertility intentions. Individuals in first-time marriages have a very strong desire to have children, while remarriage does not have a significant effect on second-child birth intentions. Income and number of siblings have a substantial effect on fertility intentions: The higher the economic level of a family, the more it can afford the cost of raising children (education, medical care, etc.); the more siblings in the family of origin, the greater an individual’s willingness to have a second child. It could be also explained by the traditional Chinese idea of “more children, more happiness.” The coefficients of social characteristics, except for health insurance, are all highly significant. Compared to western regions, individuals in the eastern and northeastern regions are more reluctant to have a second child. Those with religious beliefs are more likely to want to have a second child, probably because many religions have fertility concepts that encourage childbearing. For example, the Islamic faith opposes birth control and abortion, and Buddhism promotes the flourishing of incense. Non-agricultural hukou are only 0.968 times as willing to have subsequent children as agricultural hukou, possibly because traditional agriculture is more labor-intensive, and rural patriarchal attitudes favor sons over daughters.

**TABLE 8:** Logistic full-sample regression results.

	Variable	Coefficient	Exp(β)	Significance
Individual characteristics	Gender (Female)			
	Male	0.029	1.029	
	Age	0.005	1.005	***
	Ethnicity (Other ethnicities)			
	Han	0.039	1.040	**
	Health	–0.009	0.991	
	Education (Illiterate)			
	Primary	0.022	1.022	
	Junior_high	–0.002	0.998	
	High	–0.036	0.965	**
	Bachelor	–0.009	0.991	**
	Master and above	0.021	1.021	
Family characteristics	Marital status (No spouse)			
	First_married	0.052	1.053	***
	Remarried	–0.013	0.987	
	Family income	0.024	1.024	***
	Number of siblings	0.041	1.042	***
Social characteristics	District (West)			
	East	–0.038	0.963	***
	Central	0.136	1.146	***
	Northeast	–0.137	0.872	***
	Religion (No religion)			
	Has religion	0.018	1.018	***
	Insurance (No insurance)			
	Has insurance	0.007	1.007	
	Hukou (Agricultural hukou)			
	Non-agricultural hukou	–0.033	0.968	*
	Observations	6732		

*Significance is indicated by different numbers of asterisk: *p < 0.1, **p < 0.05, ***p < 0.01.

#### Logistic Regression by Gender

The logistic regressions by gender are shown in Table 9. Although most of the regression results are similar to those of the entire sample, they differ in some ways. For example, among the male group, members of the Han ethnic group are more willing than other ethnic groups to have a second child. Females with a high school education are less likely to have a second child, but males are not. Also, among the first-marriage group, males prefer to have a second child. Females with religious beliefs and males with non-agricultural hukou demonstrate a stronger desire to have second children. Therefore, hypothesis 4 is verified—the factors have different effects on men’s and women’s fertility intentions.

**TABLE 9:** Logistic regression results by gender.

	Variable	Coefficient	Exp(β)	Significance	Coefficient	Exp(β)	Significance
			
		Female			Male		
Individual characteristics	Age	0.004	1.004	***	0.005	1.005	***
	Ethnicity (Other ethnicities)						
	Han	–0.014	0.986		0.053	1.054	***
	Health	–0.003	0.997		0.004	1.004	
	Education (Illiterate)						
	Primary	0.021	1.021		0.023	1.023	
	Junior_high	0.004	1.004		0.000	1.000	
	High	–0.064	0.938	**	–0.005	0.995	
	Bachelor	–0.034	0.967		0.004	1.004	
	Master and above	0.019	1.019		0.015	1.015	
Family characteristics	Marital status (No spouse)						
	First_married	0.033	1.034		0.065	1.067	***
	Remarried	0.021	1.021		–0.025	0.975	
	Income	0.028	1.028	***	0.023	1.023	**
	Number of siblings	0.048	1.049	***	0.031	1.031	***
Social characteristics	District (West)						
	East	–0.027	0.973		–0.043	0.958	***
	Central	0.130	1.139	***	0.143	1.154	***
	Northeast	–0.164	0.849	***	–0.121	0.886	***
	Religion (No religion)						
	Has religion	0.035	1.036	***	0.005	1.005	
	Insurance (No insurance)						
	Has insurance	0.054	1.055		–0.023	0.977	
	Hukou (Agricultural hukou)						
	Non-agricultural hukou	–0.023	0.977		0.094	1.099	**
	Observations	2295			4437		

*Significance is indicated by different numbers of asterisk: *p < 0.1, **p < 0.05, ***p < 0.01.

## Conclusion

Compared with other studies, this paper is the first to systematically identify the influence of second-child fertility intentions in China through machine learning and logistic regression methods. Based on the 2017 and 2018 CGSS data, this paper systematically examines the factors influencing intentions for a second child from three dimensions: individual characteristics, family characteristics, and social characteristics. Three machine learning methods—ANN, RF, and XG-boost—are used to systematically screen and cross-validate the influencing factors, and a logistic regression model is used to empirically analyze the influence strength of the factors.

This paper is innovative in finding that age is the most important factor influencing the intention to have a second child, and the intention becomes stronger with age. This result contradicts previous findings that suggest that second-child policies are more likely to increase fertility intentions among younger cohorts (Meng and Lyu, 2021). Economic factors have been the focus of research (Zhou and Guo, 2020), and this study demonstrates that higher family income increases second-child fertility intentions, the results of the logistic regression are statistically significant. However, the research shows that family income is only the sixth most important in both RF and XG-boost models of all variables. In other words, economic factors are not decisive as expected.

Another breakthrough result in this study is that the larger the number of siblings, the stronger the intention to have a second child. Although the role of siblings has been discussed previously in the literature (Lan, 2021; Zhang C. et al., 2021), the results of the machine-learning model show that it ranks second and is higher than health, education, and family income. At the same time, as the current literature has been lacking a regional comparison, this paper further confirms that district has an impact on second-child fertility intentions. Like the study by Chen et al. (2019), we paper find the same result those who are healthier or have lower health risks will have higher fertility intentions for a second child.

This paper considers gender differences as well. The relationship between education and fertility has been discussed in the existing literature (Zhou and Guo, 2021) and the results of the model show that education is in the top five of all factors. This paper uses logistic regression to further reveal that women with high school and university degrees are less willing to have a second child. The current literature is less designed for men, but this paper finds that men who are married and have a non-agricultural hukou are with higher intention to have a second child. In addition, religious beliefs may increase women’s fertility intentions.

In short, this study suggests that future fertility intentions should be explained more through the interaction of individuals with their family of original and their geographical areas, rather than concentrating too much on economic factors. It also provides implications for future governmental demographic stimulus policies: traditional family policies (e.g., tax deduction, cash transfer) may not have the desired effect, and policymakers should focus more on family and socio-cultural orientations, and take into account regional and gender differences.

Although this paper utilizes the latest CGSS data and adopts a machine learning approach, it still has some shortcomings, and there is room for improvement in the future. First, the feature variables found in this paper (e.g., family policy and childcare service) may still be insufficient because many factors affect the intention to have a second child. Second, the time span is not long enough. The implementation of the second-child policy started in 2016, so the relevant data are not abundant, and most of them are cross-sectional data. Third, models of machine learning are frequently updated, and this paper only considers mainstream learning approaches. Therefore, a potential improvement in future research can be applied from these three perspectives.

## Data Availability Statement

The original contributions presented in the study are included in the article/Supplementary Material, further inquiries can be directed to the corresponding author/s.

## Ethics Statement

The studies involving human participants were reviewed and approved by National Survey Research Center at Renmin University of China, NSRC. Written informed consent to participate in this study was provided by the participants’ legal guardian/next of kin.

## Author Contributions

ML designed the study, processed the data, and drafted the original manuscript. XX provided the data and revised the manuscript. Both authors critically reviewed and approved the final manuscript.

## Conflict of Interest

The authors declare that the research was conducted in the absence of any commercial or financial relationships that could be construed as a potential conflict of interest.

## Publisher’s Note

All claims expressed in this article are solely those of the authors and do not necessarily represent those of their affiliated organizations, or those of the publisher, the editors and the reviewers. Any product that may be evaluated in this article, or claim that may be made by its manufacturer, is not guaranteed or endorsed by the publisher.
